# Prevalence of bullying in academic medical libraries in the United States

**DOI:** 10.5195/jmla.2026.2244

**Published:** 2026-07-01

**Authors:** Todd Allen Lane, Maggie Albro, Carrie S. Forbes

**Affiliations:** 1 toddlane918@gmail.com, Assistant Librarian, Research and Instruction, Buley Library, Southern Connecticut State University, New Haven, CT; 2 malbro@utk.edu, Assistant Professor and Agriculture and Natural Resources Librarian, University Libraries, University of Tennessee, Knoxville, TN; 3 forbesc@ecu.edu, Liaison Librarian to the College of Nursing Undergraduate Programs, Laupus Health Sciences Library, East Carolina University, Greenville, NC

**Keywords:** Hospital Librarians, Medical Librarian, Librarians, Health Science Librarian, Workplace Bullying, S-NAQ, prevalence

## Abstract

**Objective:**

Previous research has found bullying present across the library profession, with documented instances in school, public, and university literature, but not in academic medical and hospital library literature. This study aims to determine the prevalence of workplace bullying among academic medical and hospital librarians.

**Methods:**

An anonymous survey was distributed to members of the Medical Library Association, the Association of Academic Health Sciences Libraries, and the Association of College & Research Librarians Health Sciences Interest Group containing the Short Negative Acts Questionnaire. Quantitative analysis was conducted using the Real Statistics Resource Pack software (Release 8.9.1).

**Results:**

One-hundred eighteen survey responses met inclusion criteria. 49.2% of the sample met the threshold for self-identifying as victims of workplace bullying. This was significantly higher than the prevalence among the general librarian population (40.1%) in 2017 (p = 0.029) and consistent with findings in 2022 (51.8%; p = 0.31). More participants perceived themselves as not experienced bullying (69%) than the prevalence rate found. Those who perceived themselves as victims identified colleagues as the most frequent perpetrators (35%), followed immediate superiors (22%) and other superiors/managers within the organization (25%).

**Conclusions:**

This study showed nearly half (49.2%) of academic medical and hospital librarians included in the sample experience bullying. These findings highlight the need for an increased understanding of bullying behaviors, education on prevention, and for library leaders to develop interventions to mitigate bullying in their institutions.

## INTRODUCTION

The prevalence of workplace bullying has been extensively explored in school, public, and university library literature, but not in academic medical and hospital library literature. The objective of this study is to identify the prevalence of incivility, conflict, and dysfunction among academic medical and hospital librarians using the Short Negative Acts Questionnaire (S-NAQ). The S-NAQ is a validated tool with equivalent properties to the full Negative Acts Questionnaire and is used to measure severe and occasional bullying [1].

The Workplace Bullying Institute [WBI] definition of bullying was used for this study. The WBI defines bullying as “[r]epeated, health-harming mistreatment by one or more employees of an employee: abusive conduct that takes the form of verbal abuse; or behaviors perceived as threatening, intimidating, or humiliating; work sabotage; or in some combination of the above” [2]. Reported instances of workplace bullying are increasing in the United States [3]. In many cases, this is due to characteristics found within the work environment, such as the absence of psychological safety.

Psychological safety in the workplace significantly lessens the prevalence of bullying [4, 5]. Environments characterized by psychological safety are those in which employees feel comfortable bringing their authentic selves to the workplace, taking risks, expressing ideas, voicing concerns, and admitting mistakes without fear of retribution [3, 6, 7]. Research demonstrates that employees working in environments characterized by low psychological safety are more likely to report being bullied [4]. In 2024, 15% of respondents to the Work in America Report identified as employees of a workplace lacking psychological safety and stated negative effects of this condition spilled over to observers, colleagues, family, and personal life [3, 6–10].

While 15% of the general population reported being part of a workplace lacking psychological safety, the percentage of librarians who report working in similar environments is significantly higher [7]. In a nationwide survey of librarians conducted in 2017 by Henry et al., 40.1% of respondents reported being bullied and 59% reported witnessing bullying [11]. A post COVID lockdown follow-up study by the same researchers showed an 11% increase in bullying over a five-year period [12]. Participants in the follow-up survey identified multiple plausible factors for increased bullying, including a decrease in emotional intelligence related to social skills, self-awareness, and empathy [12]. These two studies highlight factors contributing to bullying in libraries including weak leadership, lack of communication or poor communication, and dysfunction between departments/divisions, rigid institutional hierarchy, and the negative psychological effects of COVID lockdowns, mirroring the results of studies conducted by Kisamore that established the moderating effects social competencies can play in the face of bullying [11–13].

Beyond interpersonal contributors, structural factors present in the library workplace enhance the likelihood of bullying becoming normalized. Library work carries a high demand in terms of service expectations and employee capabilities while operating with increasingly scarce resources [14]. Library work has become increasingly decentralized despite operating with multilayered administrative hierarchies [15–16]. When resource scarcity is combined with inaccessible administrators (or administrative decision-making clouded by multiple layers of hierarchy) and lack of transparency between operating units, distrust forms between workers who need to compete for resources and those who allocate them. The resulting low trust toward institutional leaders combines with high stress in everyday work, a combination which has been demonstrated to lead to rapid acceptance of bullying as a mechanism for survival [14]. These factors are further compounded in academic workplaces, such as universities, which exhibit a higher-than-average degree of workplace bullying [9, 14, 16–19]. The same is true for medical settings, particularly hospitals, where the crisis of workplace bullying has been widely explored [20–22]. Despite the understanding of bullying as a phenomenon in the profession and within these two specific work settings, research has not yet explored the unique experience of librarians working within medical settings.

Across the United States workforce, the targets and perpetrators of bullying generally fall into certain categories. Women tend to be targets more than men. Racial minorities are almost exclusively targeted more than white workers, with Hispanic populations consistently experiencing the highest rates of bullying among all racial groups [23]. Recent library-specific studies have not observed these demographic differences in bullying; however, this discrepancy may be due to a largely heterogeneous library workforce that has not employed adequate sampling of non-majority individuals [14, 16]. Over the past ten years there has been an increase in supervisors perpetrating bullying across the general population, with a 2022 survey of librarians indicating roughly 70% of targets experienced bullying at the hands of their supervisor [23].

The heightened prevalence of bullying in libraries has consequences for workplaces and employees alike. The psychological effects of bullying on the workplace environment include decreased team and employee engagement, increased costs, toxic workplace culture, decreased institutional reputation, and increased employee intent to leave and staff turnover [3, 24]. Personal impacts experienced by the target include burnout, physical health impacts, emotional health impacts, decreased motivation, lower self-esteem, feelings of powerlessness, PTSD, and suicide in extreme cases [3, 10, 24–31]. The psychological impacts of bullying experienced by the target oftentimes spill over to team members who witness bullying.

Although Second Victim Phenomenon is closely linked with emotional distress following witnessed medical trauma, this concept may help explain the psychological and professional impact of workplace bullying by affiliated staff not directly involved in patient care who may experience the impact of these events beyond the clinical arena [32]. Librarians in hospitals or academic medical settings that directly serve clinical staff may witness or be a target of bullying or workplace aggression resulting from the Secondary Victimhood of medical practitioners. Witnessing adverse events, including bullying, erodes workplace psychological safety and contributes to decreased mental health, sleep, increased absenteeism, self-esteem issues, feelings of rage/despair, and other health problems among team members, much like they do the individual directly subjected to the adverse event [3, 10, 25–28]. Additionally, witnesses may fear repercussions surrounding an attempt to intervene, report, or stop bullying [10, 28, 33, 34]. Employees who witness bullying often report the breakdown in trust of colleagues and leadership, with the breakdown of trust often has a ripple effect on the team.

More recently, Rosander proposed the concept of “the blast radius” of bullying to explain how deep the effects of witnessing bullying reach into a team [7]. The concept of a blast radius of bullying uses social information processing theory – which provides a framework for how people interpret information in social settings they participate in and observe – to identify the ripple effect of bullying on the physical, social, and emotional wellbeing of those who witness mistreatment. Rosander found that the negative effects of bullying reach far enough to negatively impact the health, wellbeing, and job attitudes of those who witness abuse thus making them co-victims of workplace violence [7].

## METHODS

The Short Negative Acts Questionnaire (S-NAQ) is a validated 9-item inventory used to determine exposure to workplace bullying. The S-NAQ has equivalent properties to the full Negative Acts Questionnaire (NAQ) used to measure severe and occasional bullying.[1] While there are multiple studies defining score ranges to different “levels” of bullying, there has yet to be a study specific to the United States population [35–37]. Survey scores were divided by the criteria ranges established in the S-NAQ Danish cohort study, with “not being bullied” defined as a score of <12, experiencing “occasional bullying” as a score between 12-15, and experiencing “severe bullying” as a score of >=16 [36]. Sample scores on the S-NAQ ranged from 9 (the lowest possible score) to 45 (the highest possible score).

The S-NAQ was placed in a Qualtrics survey, which began with an informed consent statement and concluded with questions relating demographic information. The platform was configured to calculate the S-NAQ score for each participant upon the completion of the survey. To ensure respondents met the eligibility criteria, screening questions for each criterion were placed at the beginning of the survey. Participants were eligible to participate if they were over the age of eighteen, were employed or retired from an academic medical or hospital library in the United States, and held an MLIS or equivalent degree; however, students of any kind were not eligible to participate. If a respondent answered screening questions to indicate they did not meet all eligibility criteria, they would be thanked for their time and the survey would close.

The survey was distributed by email to membership-wide listservs of the Medical Library Association (MLA), Association of Academic Health Sciences Libraries (AAHSL), and the Association of College & Research Librarians (ACRL) Health Sciences Interest Group (HSIG) listservs. The survey was distributed twice, on October 31 and November 20, 2024, and was open between October 31, 2024, and November 30, 2024. Members of MLA, AAHSL, and ACRL HSIG active on their organizations’ listservs who met the eligibility criteria were invited to participate upon consent.

The Real Statistics Resource Pack software for Microsoft Excel was used to analyze survey results (Release 8.9.1) [38]. Analysis included descriptive statistics of all demographic variables and summary statistics of S-NAQ scores. Welch’s analysis of variance (ANOVA) was selected as the method of analyzing differences in S-NAQ scores between participants in different categories of age, gender, sexual orientation, duration of career, or duration in the current role (respectively). Welch’s ANOVA was selected due to the lack of homogeneity of variance in all categories required for a standard single factor ANOVA. One-sample proportion testing was used to situate the bullying prevalence level observed in this study against the studies conducted by Henry et al. in 2017 and 2022 by using the proportion of the samples of the previous studies as the population proportion in the analysis [11,12]. The S-NAQ has been validated as an equivalent measurement of the NAQ and is considered an appropriate comparator to the full questionnaire across studies [1].

Ethics board approval for this study was completed by each author’s institution. This study was determined exempt under Category 2 by the University of Tennessee, Knoxville Institutional Review Board (1528961), Yale University Human Research Protection Program (2000038627), and East Carolina University & Medical Center Institutional Review Board (24-001998), all of which reviewed all project documents, including the protocol, informed consent, recruitment messaging, and survey instrument.

## RESULTS

One hundred forty-two survey responses were collected. Eight responses were excluded for not meeting inclusion criteria related to employment, age, or degree status. Sixteen participants did not fully complete the S-NAQ, rendering their results invalid [1]. This resulted in a final sample size of 118.

Demographic data were collected for 115 respondents. This revealed that respondents were majority white (90%, n=104/115) and female (87%, n=100/115), aligning with the 2024 Current Population Survey of librarian demographics [39]. The majority of respondents identified as heterosexual (77%, n=88/115), demonstrating a slightly higher representation of this demographic than that found in MLA 2019 Diversity and Inclusion Task Force survey, which was distributed to the entirety of the Medical Library Association [40]. Of the respondents in the present study, 27 (23%) identified as LGBTQ+, which did not provide a statistically rigorous quantity to allow distinct analysis of the group. The sample’s age distribution aligns with the 2019 MLA Task Force findings [40].

Of the 115 respondents who answered questions regarding demographic information and their professional roles and experience, respondents included professional staff (48%, n=55), faculty with no tenure option (31%, n=36), faculty with tenure or on the tenure-track (15%, n=17), and staff employed in some other category (6%, n=7). Respondents in administrative roles included library directors (23%, n=26), managers (17%, n=19), and administrators (5%, n=6). Respondents’ ages spanned a range of categories, 3% (n=3) between the ages of eighteen and twenty-four, 16% (n=18) between twenty-five and thirty-four, 26% (n=30) between thirty-five and forty-four, 24% (n=28) between forty-five and fifty-four, 23% (n=26) between fifty-five and sixty-four, and 9% (n=10) above the age of sixty-five. Most respondents were largely in the mid- or late stage of their career with 31% (n=36) working in the field for over 21 years.

**Figure 1:**
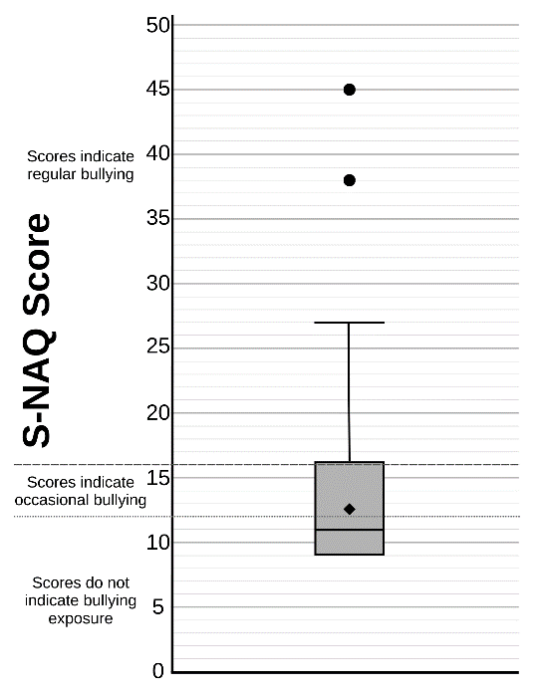
Distribution of S-NAQ scores and bullying category classification across sample group.

The mean S-NAQ score of the full respondent set (n=118) was 13.5, though the median was 11, placing the average participant experience on the edge of the classifications of not being bullied or occasionally being bullied (see fig. 1) [36]. Just above half (51%, n=60) of respondent scores indicated they did not experience workplace bullying in the past six months. Twenty-one percent (n=25) experienced occasional bullying, and 23% (n=27) experienced severe bullying. Welch’s ANOVA (Table 1) showed no differences in S-NAQ score based upon age, gender, sexual orientation, duration of career, or duration in the current role (p > .05).

**Table 1:** Welch’s Analysis of Variance of S-NAQ score by sample group demographic and career characteristic

	F-statistic	df1	df2	p-value	Significance
Age	0.412	5	19.757	0.835	No
Gender	1.534	2	7.972	0.273	No
Sexual Orientation	0.819	3	13.082	0.506	No
Duration of career	0.289	4	36.467	0.883	No
Duration in current role	0.342	4	25.205	0.847	No

Respondents reported being excluded (22%, n=26) and having information withheld that affects their performance (18%, n=21) on a monthly, weekly, or daily basis more frequently than other behaviors in the questionnaire. Other insidious forms of negative acts, such as being the subject of gossip and rumors (9%, n=11) and facing persistent criticism related to one’s work (9%, n=11), were reported on a monthly, weekly, or daily basis more often than overt behaviors like direct insults (2.5%, n=3) or being shouted at (3%, n=4). Behaviors were reported consistently across demographic factors.

Bullying prevalence among the sample was calculated by combining those who experience occasional bullying and those who experience severe bullying. This yielded 49% of the sample group experienced bullied according to S-NAQ criteria. This percentage is consistent with levels noted among the librarian profession as a whole (52%) in 2022 (p = 0.31) [12]. Consistent with the level among the general profession in 2022, the percentage from this study sample was significantly higher than that observed (40%) in 2017 (p = 0.029) [11].

There was no statistically significant difference in demographics between the groups who were bullied and those who were not; however, observational differences in some respondent categories were seen by the researchers and suggest further study with a larger dataset is needed. All respondents between eighteen and twenty-four years of age and two-thirds of those age sixty-five and older received scores showing they experienced bullying. In all remaining age bands, there was a relatively even divide between those who had been bullied and those who had not. No observed differences were seen in gender or sexual orientation between bullied and non-bullied respondents. While most career duration ranges were split relatively evenly, two-thirds of the respondents in their fourth through seventh years working in the library field appeared in the non-bullied group. Twice as many of those in the upper middle ranges of duration in their current role (ranging from eight to twenty years) appeared among the bullied than the non-bullied.

Of significance, more participants (69%, n=81) self-identified as having not experienced bullying than indicated by S-NAQ scores while 29% (n=34) experienced occasional bullying and 2% (n=3) experienced severe bullying over the same period. Of the 37 participants who reported occasional or severe bullying, colleagues were identified as the most frequent perpetrators (57%, n=21), followed by other superiors/managers within the organization (41%, n=15) and immediate superiors (35%, n=13). Subordinates were identified by 13% (n=5) of participants as their perpetrators with customers/patients/students/etc. identified by 16% (n=6) of participants. Thirty-one participants indicated they experienced bullying behavior from a mean of 2.125 female perpetrators (range: 0-6), while 19 indicated they experienced bullying from a mean of 0.840 male perpetrators (range: 0-3), and no respondents indicated non-binary/third gender perpetrators. The most commonly reported behaviors by those who self-identified as bullying victims matched the pattern shown by the full set of respondents, with insidious negative acts reported more commonly than overt forms of bullying.

## DISCUSSION

To the best of the authors’ knowledge, this is the first study to assess the prevalence of bullying experienced by academic medical and hospital librarians. Responses indicate that 49.2% of participants are considered bullied based on cumulative S-NAQ scores. This number is consistent with prevalence levels among the general librarian population [12]. Instances of bullying among medical librarians is significantly higher than those of the general workforce [8]. Although the S-NAQ does not include questions about contributing factors to bullying, post-COVID literature supports the hypothesis that heightened interpersonal, emotional, and societal factors are a key component of increases in bullying experienced after the onset of the pandemic [8, 10, 28, 11–13, 33, 34].

Academic medical and hospital librarians navigate a complex power gradient due to the intersection of hospital and academic library work cultures in their domain of practice. The hierarchies within medical settings limit the power of librarians, whose credentials are removed from the context of medical credentials, to communicate their needs, as well as their value, within the organization [41–43]. To complicate matters for these librarians, medical settings are known for high levels of workplace violence, including bullying, from administrators to trainees [44]. Existing in an employment power gradient with a high degree of workplace violence may account for the elevated instances of bullying among medical librarians compared to the general population.

A mismatch between S-NAQ score and self-perception of bullying victimhood was identified. The average score within the sample group falls within the ‘occasional workplace bullying’ range (M = 13.5). This does not align with participants’ views of their own experiences. Participants generally indicated that treatment they experienced did not classify as bullying, but nearly half of the sample met criteria for bullying to some degree. The disparity of perception of bullying victimhood and calculated S-NAQ score has been identified by in the past and can result from many factor [45–47]. One explanation is the subjective nature of self-labeling, as individuals differ in their personal thresholds for identifying as a victim [48, 49, 50]. Other factors could include normalization of bullying behaviors, the level of mistreatment an individual can tolerate before labeling themselves as being bullied, the use of denial or minimalization as a comping mechanism, or cultural and organizational norms. People can experience the impact of bullying without feeling victimized, meaning they experience health and psychological consequences often before they identify as a victim of the experience that led to them [45, 47].

Recent research has found that individuals who are newly exposed to workplace bullying and those who have previously identified as workplace bullying victims are impacted by bullying in the same ways; however, previous victims are more likely to self-identify as victims than their newly exposed counterparts [51]. While further research is needed to determine the exact cause of this discrepancy, Hoprekstad et al. posit that it may be due to increased recognition of bullying behavior or enhanced predisposition to ascribe negative intent that has not been built into the social processing of those who are newly exposed [51].

Another potential contributor to this disparity between self-perception and S-NAQ score can stem from the lived experience of an individual during their formative years. Individuals who experienced aggression as normal (whether that was performing or receiving the behavior) may be more disposed to accept similar behaviors as normal and expected later in their lives [52]. Similarly, within the unique context of academic medical and hospital libraries, it is possible the regular practice of workplace hostility by medical colleagues to one another tempers librarian perceptions of their own treatment within the workplace by placing it within a culture of normalized hostility [44]. Within our sample the most frequently reported behaviors were insidious (such as being excluded), making it easy to question if what one has experienced is bullying, particularly if set alongside departments with more overt negative acts, such as yelling.

Among our sample, the most frequent category of perpetrators were colleagues, which is consistent with findings of previous studies on perpetrators of workplace bullying [14, 16, 17, 53, 54]. Library work environments are team-based, regardless of the independence of specific job roles. Team-based work involves fluctuating social status among colleagues. In a work setting, social status impacts the opportunities, resources, and autonomy individual workers have access to. Within this context, colleagues can use positive or negative social behaviors to change their access and advantage relative to one another [14]. Bullying can be used either to preserve a favorable position through exerting power over another person or to weaken and disempower someone with greater advantage through targeted negative behavior. Colleagues may initially engage in less severe bullying behaviors such as rudeness or incivility; however, through cycles of escalating negative actions commonly observed in the workplace, these behaviors may become normalized as an accepted forms of interaction [10, 54].

Following colleagues, two different categories of superiors – immediate supervisors and other superiors/managers within the organization – made up the group of next most frequent perpetrators. When combined, those in leadership roles created the largest set of perpetrators among the responses (47%), aligning with findings of past library bullying studies [56]. Supervisors and managers in academic medical and hospital libraries typically serve as hybrid medical managers – a person well-credentialed within a non-medical field managing in a medical setting. These leaders face unique challenges when unsupported by human resources and other officials within their larger organization [57]. Without adequate support, input from library leaders is more limited within the boarder organizational context than that of their counterparts. As a result, libraries are typically a lower priority for budget, personnel, and operational resources. These circumstances can leave library leaders feeling disempowered while navigating and reconciling competing professional norms within their workplace. This can result in trickle-down tension within the library as those in charge struggle to connect the library within the broader organizational landscape. Library leaders are not immune to stress, and their inability to effect change and exact influence in the broader organizational landscape can intentionally or inadvertently trigger negative behavior within their own units [10, 54]. This tension might pair with the blast radius of hostility in the larger workplace to create a sense of distress in the library environment. Workplace incivility and distress become systemic in a unit when negative leadership behavior, stressful organizational culture, exposure to trauma, and insufficient support from outside the unit interact to shape the daily experience of employees [54, 56]. As such, managers and supervisors must take measures to care for their own distress to break the cycle before it becomes a pervasive issue in their library.

It is important to note that all employees in all workplaces are responsible for keeping their workplace civil. Leaders, both within the library and in the larger organization, must establish a tone for a civil workplace and hold those who engage in bullying accountable. Clearly stated policies, with definitions of unacceptable behavior, a defined process for reporting and investigation, and protection from retaliation for good-faith reporting, are essential in maintaining a civil culture. Regularly held civility, conflict management, empathy, and bystander intervention training for all employees reinforces and reminds everyone of expectations for workplace behavior [11]. In medical settings, support from institutional leaders and human resources can offset power imbalances that isolate and disadvantage librarians to further support an anti-bullying culture [57]. While there is no one size fits all solution to workplace bullying, consistent awareness, clear communication, and organizational involvement can be combined to work towards civil work interactions for all.

### Limitations and Further Research

Self-report surveys are susceptible to response bias. Research supports the threat of response bias to the validity of findings of self-report surveys on negative and subjective subject matters [55]. To account for this, the researchers chose to include the WBI definition of bullying in the survey and to use the abbreviated version of the Negative Acts Questionnaire to reduce the number of items that might elicit misunderstanding or negative emotions. As the survey instrument has been previously validated and deemed reliable in the English-language translation, it is believed that the validity and reliability of the survey method remains in-tact and an appropriate venue for making confident conclusions.

The small sample size with an inexact response rate limits generalizability. At the time of survey, MLA membership was stated to be over 2,500, AAHSL documents 170 member institutions based in the United States, and ACRL Health Sciences Interest Group membership was between 1,000 and 2,000 individuals [58–60]. This resulted in the possibility of overlap in membership between the three groups and the inability to account for how many members participate in official listservs. As a result, it was not possible to calculate exact sample size, nor an accurate response rate. Additionally, association memberships can be cost prohibitive and the ability to reach potential participants without individual or organizational access to their affiliated listservs limits the participant pool. This introduces an additional limitation through the inability to assess how representative the sample is of the academic medical and hospital librarian community. Generalizability of the findings is further limited by the use of the English-language translation and geographic distribution of the study, making these findings unsuitable for reference beyond an English-speaking United States population.

A limitation of announcing the survey availability through larger library associations and groups that contain members outside of the sample population being sought (in this case, all health sciences librarians) is the potential for ineligible respondents to seek participation. Although screening questions were placed before the start of data gathering for the study, there is a chance of false reporting by participants that could allow inclusion where it should not be present.

Further research is needed to explore the characteristics of both victims and perpetrators in this subset of librarians. While this study explored academic medical and hospital librarians as one group, workplace dynamics in the two types of organizations can vary widely. Continued study is needed to explore if there are differences in bullying between different types and sizes of libraries, among other contextual workplace factors. Additionally, more research is needed to develop insight into how members of societal outgroups experience bullying in a different way than their majority colleagues [19].

## CONCLUSIONS

Our analysis of collected S-NAQ demonstrates 49.2% of respondents experienced some form of workplace bullying in the past 6 months. The data shows a mismatch between self-identification of bullying and being bullied according to S-NAQ criteria. Our survey findings demonstrate that the rate of bullying experienced by academic medical and hospital librarians are comparable to the findings of Henry et al.’s post-COVID five-year follow-up study [1]. The results of this study highlight the need for increased understanding of bullying behaviors, education on prevention, and for library leaders to develop skills and interventions to mitigate bullying in their organizations.

## Data Availability

Data associated with this article are available in Open Science Framework at https://osf.io/ejxwd/.
