# Erratum to: Lane TA, Albro M, Forbes CS. Prevalence of bullying in academic medical libraries in the United States. J Med Libr Assoc. 2026 Jul;114(3):246–254. DOI: 10.5195/jmla.2026.2244.

**DOI:** 10.5195/jmla.2026.2612

**Published:** 2026-10-01

**Authors:** Todd Lane

Appendix A was not included with the originally published manuscript. The appendix has since been added and is now available with the article.

